# Benign mesenteric lipomatous tumor in a child: a case report and literature review

**DOI:** 10.1186/s40792-020-01020-7

**Published:** 2020-09-30

**Authors:** Naoki Hashizume, Takato Aiko, Suguru Fukahori, Shinji Ishii, Nobuyuki Saikusa, Yoshinori Koga, Naruki Higashidate, Saki Sakamoto, Shiori Tsuruhisa, Hirotomo Nakahara, Hiroko Muta, Hiroaki Miyoshi, Yoshiki Naito, Hidetaka Yamamoto, Yoshinao Oda, Yoshiaki Tanaka, Minoru Yagi

**Affiliations:** 1 Department of Pediatric Surgery, Kurume University School of Medicine, 67 Asahi-machi, Kurume, Fukuoka 830-0011 Japan; 2 Department of Diagnostic Pathology, Kurume University School of Medicine, Asahimachi 67, Kurume, 830-0011 Fukuoka Japan; 3 Department of Anatomic Pathology, Graduate School of Medical Sciences, Kyushu University, Higashku umade 3-1-1, Fukuoka, 812-8582 Fukuoka Japan; 4 Division of Medical Safety Management, Kurume University Hospital, Asahimachi 67, Kurume, 830-0011 Fukuoka Japan

**Keywords:** Benign mesenteric lipomatous tumors, Lipoma, Children, Lipoblastoma

## Abstract

**Background:**

Lipomatous tumors are the most common type of soft-tissue tumors. Benign lipomatous tumors are lipomas and lipoblastoma. We herein report a case of benign mesenteric lipomatous tumor and the largest collection of known benign mesenteric lipomatous tumors in children in the literature.

**Case presentation:**

A 3-year-old girl presented with repeated dull abdominal pain and left abdominal mass swelling. On a physical examination, the child had a soft, moderately distended left abdomen that was not tender when palpated. Computed tomography and magnetic resonance imaging demonstrated a large fatty mass within the mesentery, measuring approximately 8 × 6 cm. The mass extended from the right upper quadrant to the lower pole of the kidneys. Laparotomy with resection of the mesenteric tumor was performed under general anesthesia. A well-capsuled tumor was a soft, yellow mass and found loosely attached to the mesenterium of the ileum. A histopathological examination demonstrated the lobular proliferation of mature adipocytes. Atypical lipoblasts were not seen. These features are compatible with benign lipomatous tumor, such as lipoma or lipoblastoma with maturation.

**Conclusion:**

In conclusion, benign mesenteric lipomatous tumors tend to be large in size over 10 cm in longitudinal length. However, resection is well tolerated in the vast majority of cases with benign post-operative courses.

## Introduction

Lipomatous tumors are the most common type of soft-tissue tumors. Benign lipomatous tumors are “lipomas” and “lipoblastoma”. Lipoma is a rare benign lesion of mature adipose tissue. It is a well-defined, noninvasive, and encapsulated mass that can be discovered in asymptomatic patients or may cause variable nonspecific symptoms, depending on its size and location [1, 2]. Lipoblastoma is a rare benign soft-tissue tumor that occurs most commonly in infants and children [3, 4]. The vast majority are detected in children under 3 years old, with over 80% of cases occurring before 3 years old and 40% before 1 year old [3–7]. These tumors occur commonly in the extremities, trunk, head, and neck. However, benign mesenteric lipomatous tumors are rare. The long-term prognosis for lipoblastoma is usually excellent [5]. Metastases have never been reported, but the recurrence rates have been reported to range from 9 to 22%, which is attributed to incomplete initial excision of the tumor [6, 7]. Mesenteric lipomatous tumors are slow-growing, mobile, soft masses that do not infiltrate the surrounding organs. Gastrointestinal tract lipomas mostly present as an insidious-growing, soft, mobile mass without penetration into the surroundings.

We herein report a case of benign mesenteric lipomatous tumor and the largest collection of known benign mesenteric lipomatous tumors in children in the literature.

## Case report

A 3-year-old girl presented with repeated dull abdominal pain and left abdominal mass swelling. There were no evident congenital abnormalities at birth nor any familial history of disease. On a physical examination, the child had a soft, moderately distended left abdomen that was not tender when palpated. The hemoglobin, alphafetoprotein, and beta-hCG levels were normal.

**Fig. 1:**
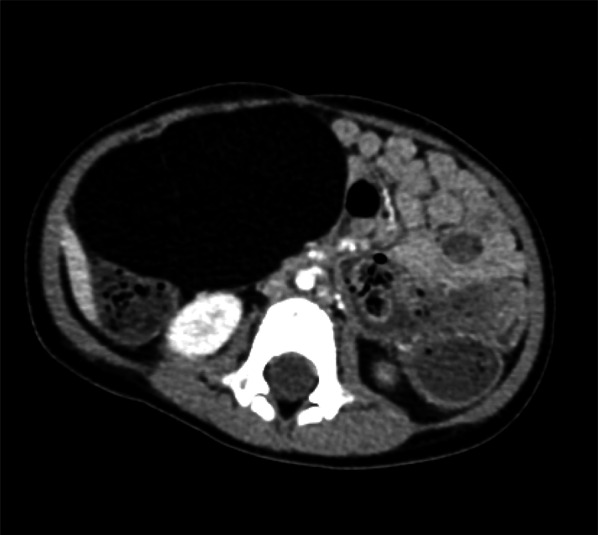
Computed tomography obtained a large low-density mass within the mesentery, measuring approximately 8 × 6 cm

**Fig. 2:**
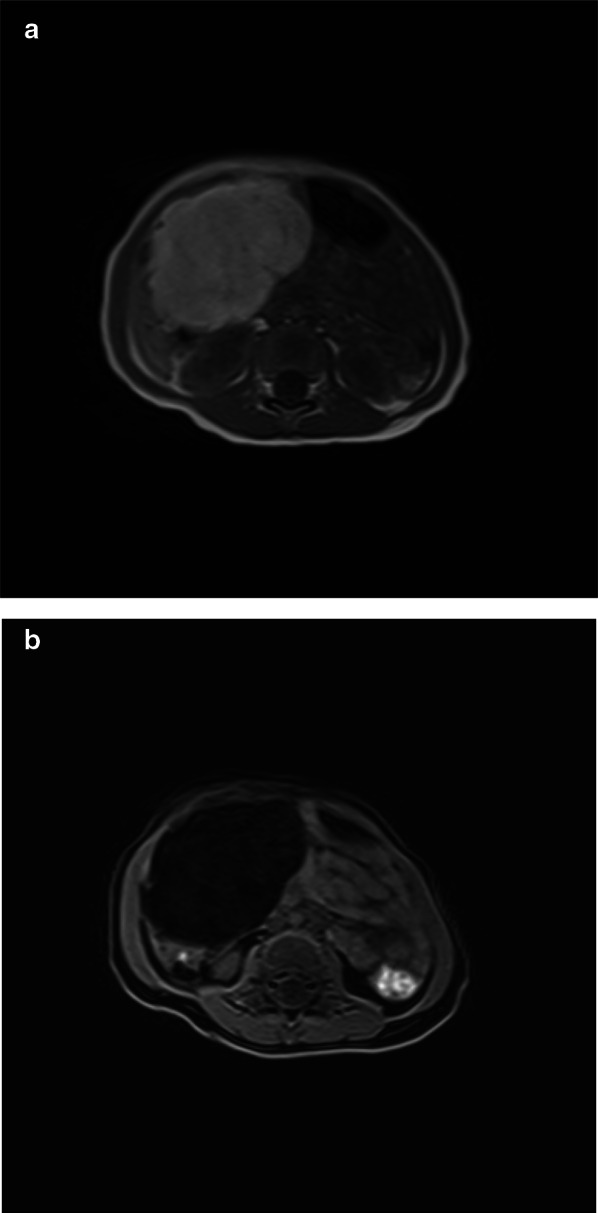
Magnetic resonance imaging coronal section image obtained a well-encapsulated soft-tissue mass lesion with high intensity in the mesenteric region, **a** T1-weighted image obtained the mass lesion with high intensity signal. **b** Fat suppression inversion recovery imaging obtained an interposing fat component showing a reduced signal

Abdominal ultrasound showed a heterogeneous soft-tissue mass measuring φ8 cm. Computed tomography demonstrated a large fatty mass within the mesentery, measuring approximately 8 × 6 cm. The mass extended from the right upper quadrant to the lower pole of the kidneys (Fig. 1). Magnetic resonance imaging (MRI) revealed a well-encapsulated soft-tissue mass lesion in the mesenteric region. The mass had a clearly defined margin and a reticular pattern with an interposing fat component showing a reduced signal on fat suppression inversion recovery imaging (Fig. 2a, b). Based on these findings, the mass was suspected of being a benign soft-tissue tumor, most likely lipoma or lipoblastoma.

**Fig. 3:**
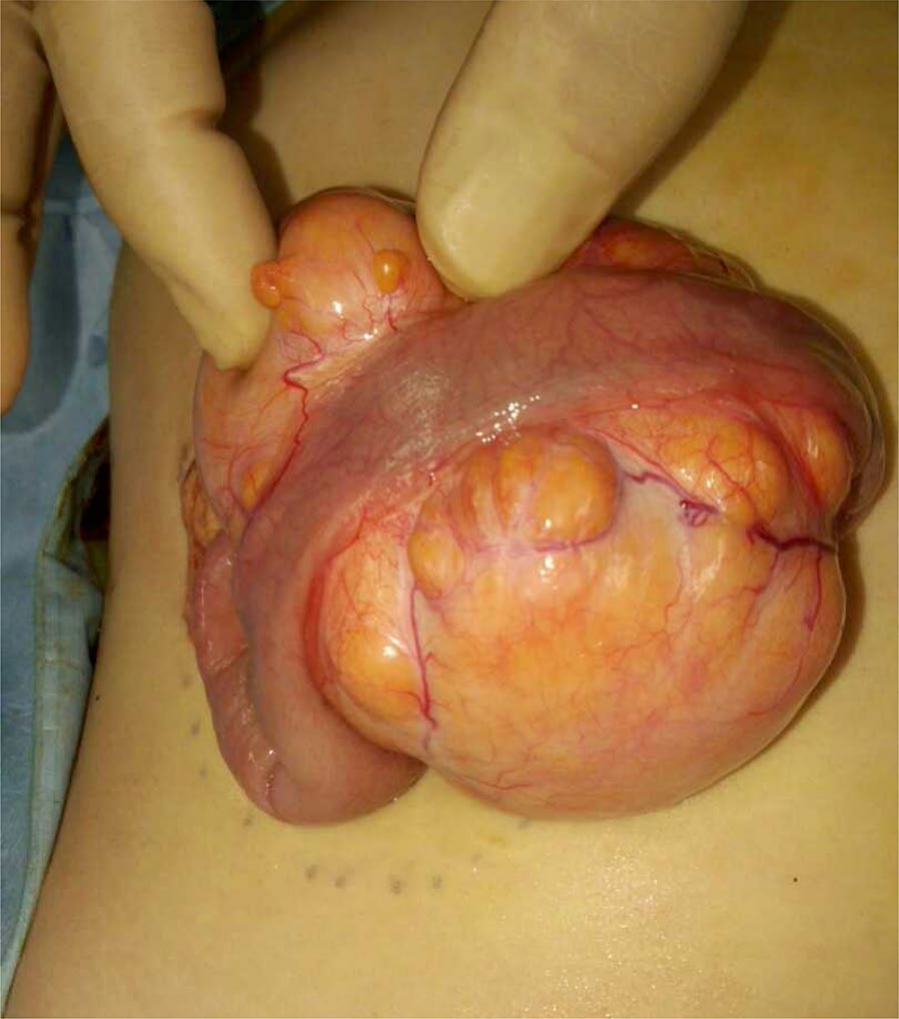
A well-encapsulated tumor measuring 8 × 6 cm was found loosely attached to the mesenteric tumor

Laparotomy with resection of the mesenteric tumor was performed under general anesthesia. A well-capsuled tumor was a soft, yellow mass and found loosely attached to the mesenterium of the ileum. The tumor was located 6 cm from the ileocecal valve. The tumor was not separated from small intestine. Tumor was resected with small intestine which was 5 cm and end-to-end anastomosis of the small intestine was performed without injury to adjacent structures (Fig. 3). The tumor was well circumscribed with a thin, fibrous capsule and a yellow, lobulated fatty parenchyma separated by thin fibrous septa with punctate vessels.

**Fig. 4:**
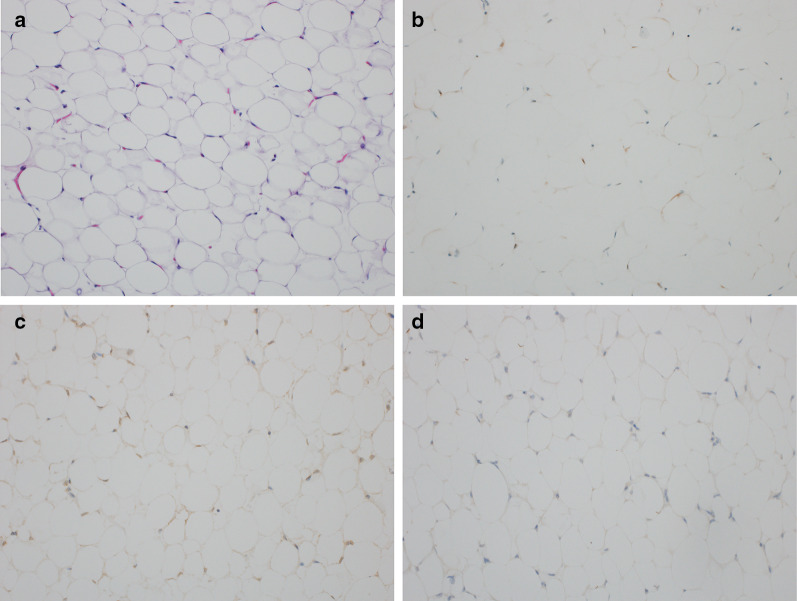
A histopathologic examination revealed the tumor cells demonstrated the lobular proliferation of mature adipocytes (**a**). Immunohistochemistry results were as follows: adipocytic cells positive for p16 (**b**), positive for cyclin-dependent kinase 4 (**c**), and negative for murine double minutes (**d**)

A histopathological examination demonstrated the lobular proliferation of mature adipocytes. Atypical lipoblasts were not seen (Fig. 4a). Immunohistochemically, there were adipocytic cells positive for p16 and cyclin-dependent kinase 4 (CDK4) but negative for murine double minutes (MDM2) (Fig. 4b–d). The feature is compatible with benign lipomatous tumor, such as lipoma or lipoblastoma with maturation.

Follow-up at 2 years revealed no evidence of recurrence.

## Discussion

Mesenteric lipomatous tumors are slow-growing, mobile, soft masses that do not infiltrate the surrounding organs. Ultrasound can detect abdominal lipomatous tumors as homogenous echogenic masses; however, they may present a confusing picture, from a homogenous to a heterogeneous echo pattern [1]. According to the computed tomography findings, other fat attenuation processes associated with the mesentery are considered as differential diagnoses, including liposarcoma, lymphangiomas, and cavitating mesenteric lymph node syndrome [8]. MRI has the highest sensitivity for the pathology of the tumor among imaging modalities, as the increased vascularity in lipoblastomas compared to lipomas showed a low intensity on T1-weighted images [6]. MRI is, therefore, the current recommended modality for follow-up, particularly in cases of incomplete resection or prior recurrence. Resection and a pathological examination are ultimately needed to make a definitive diagnosis.

According to the pathological findings, the maturation pattern of lipoblastoma tends to be characterized by a high proportion of mature adipocytes at the center of the lobule with lipoblasts on the periphery. Some reports suggest that lipoblastoma may spontaneously mature or regress [4]. Coffin et al. reported the maturation of an incompletely excised lipoblastoma into a mature lipoma [5]. Similar to the present case, although atypical lipoblasts were not detected, it was difficult to distinguish between lipoblastoma with maturation and lipoma give the patient’s age.

The recent use of cytogenetics has proven useful for making the diagnosis, as translations involving the long arm of chromosome 8, particularly 8q11-13, with or without pleomorphic adenoma gene 1 (PLAG1) oncogene rearrangements, have been found to be associated with lipoblastomas [7–9]. This rearrangement targets the PLAG1 gene and has been reported in 82% of lipoblastomas but only 3% of conventional lipomas and never in myxoid liposarcoma [9]. In the present case, we could not examine if there was PLAG1 oncogene rearrangements or not. As atypical lipoblasts were not seen, there was, therefore, no need to distinguish lipoma from lipoblastoma with maturation.

The immunohistochemical trio of CDK4, MDM2, and p16 is a useful ancillary diagnostic tool that provides strong support for distinguishing differentiated liposarcoma from other adipocytic neoplasms [10]. A previous study found that MDM2 is highly sensitive for atypical lipomatous tumor, and without taking this marker into account, there has been a tendency to misclassify atypical lipomatous tumors as lipomas in the past [11].

A literature search was performed using the electronic database “PubMed” for all patient reports in the English literature with benign mesenteric lipomatous tumors using the search term “mesenteric lipoma”, “mesenteric lipoblastoma”, and “benign mesenteric lipomatous tumor”. Relevant data were extracted from all primary reported patients. Patients included in multiple reports were used only once for the analysis. All patients under 15 years old were combined to create this report. The clinical features of the current case were consistent with those previously reported, including her age, gender, onset, size and location of tumor, preoperative diagnosis, pathological diagnosis, operation, and complications.

All patient data from 1956 to 2020 were combined to create this report [4, 12–45]. There have been 44 cases of mesenteric lipoma and lipoblastoma in children, as shown in Table 1. These patients were 18 males and 18 females excluded cases which were not reported about gender. Twenty cases were lipoma, and 24 cases were lipoblastoma. The age at the presentation ranged from 5 months to 11 years old. Especially in the cases with lipoblastoma, the age at the presentation in 17 cases (70.9%) was under 3 years old. The tumors ranged in longitudinal length from 8 to 31 cm. Almost all tumors were over 10 cm in longitudinal length excluded not reported cases. Lipomas were located on the mesentery of the small intestine (50.0%) or omentum (10.0%). On the other hand, lipoblastomas were located on the mesentery of the small intestine (33.3%), omentum (12.5%), jejunum (4.16%), colon (8.3%), and in the peritoneal cavity (4.16%). The other reports did not describe the retroperitoneal side in detail. About the onset of cases with lipoma, 13 cases (65.0%) had abdominal pain. On the other hand, 8 cases (33.3%) with lipoblastoma had abdominal pain. Because over 50% of the cases with lipoblastoma were under 3 years old, they could not complain of abdominal pain. In 26 cases, the operative procedures were reported. In 15 cases, tumor resection without anastomosis was performed, while 11 cases received tumor resection with end-to end anastomosis. There were no cases of incomplete resection excluding 8 cases where the details were not reported. In 9 cases, the tumors weighed over 1 kg. Six cases presented with volvulus and bowel obstruction. There were no cases of recurrence and post-operative complications.

**Table 1:** Summarized cases of lipoma and lipoblastoma tumor in children of the literatures

Pathological diagnosis	Lipoma	Lipoblastoma
Case, *n*	20	24
Sex		
Male	7 (35.0)	11 (45.8)
Female	7 (35.0)	11 (45.8)
NR	6 (30.0)	2 (8.3)
Age, *n* (%)		
0–6 months	0 (0.0)	1 (4.16)
6–12 months	4 (20.0)	4 (16.7)
1–3 years	4 (20.0)	12 (50.0)
Over 3 years	6 (30.0)	6 (25.0)
NR	6 (30.0)	1 (4.16)
Onset, *n* (%)		
Abdominal distension	7 (35.0)	8 (33.3)
Abdominal mass	4 (20.0)	11 (45.8)
Abdominal pain	13 (65.0)	8 (33.3)
Vomiting	4 (20.0)	6 (25.0)
Appetite loss	1 (5.0)	1 (4.16)
NR	1 (5.0)	1 (4.16)
Tumors ranged in longitudinal length		
Mean ± SD (cm)	18.4 ± 6.6	14.1 ± 3.7
Over 10 cm, *n* (%)	13 (65.0)	14 (33.3)
Under 10 cm, *n* (%)	0 (0.0)	1 (4.16)
NR, *n* (%)	7 (35.0)	9 (37.5)
Location, *n* (%)		
Mesentery of the jejunum	0 (0.0)	1 (4.16)
Mesentery of the small intestine	10 (50.0)	8 (33.3)
Mesentery of the colon	0 (0.0)	2 (8.3)
Omentum	2 (10.0)	3 (12.5)
Peritoneal cavity	0 (0.0)	1 (4.16)
NR	8 (40.0)	9 (37.5)
Weight, *n* (%)		
Under 1 kg	4 (20.0)	3 (12.5)
Over 1 kg	4 (20.0)	5 (20.8)
NR	12 (60.0)	16 (66.7)
Volvulus, *n* (%)		
Yes	3 (15.0)	3 (12.5)
No	12 (60.0)	17 (70.8)
NR	5 (25.0)	4 (16.7)
Operation, *n* (%)		
Only resection	9 (45.0)	6 (25.0)
End-to-end anastomosis	4 (20.0)	7 (29.2)
NR	7 (35.0)	10 (41.7)
Complete resection, *n* (%)		
Yes	20 (100)	16 (66.6)
No	0 (0.0)	0 (0.0)
NR	0 (0.0)	8 (33.3)

*NR* Not reported, *SD* standard deviation

## Conclusion

In conclusion, benign mesenteric lipomatous tumors tend to be large in size over 10 cm in longitudinal length. However, resection is well tolerated in the vast majority of cases with benign post-operative courses.

## Data Availability

All data generated during this study are included in this published article.

## Abbreviations

MRI: Magnetic resonance imaging
CDK4: Cyclin-dependent kinase 4
MDM2: Murine double minutes
PLAG1: Pleomorphic adenoma gene 1
